# Sustainable Synthesis of the Active Pharmaceutical Ingredient Atenolol in Deep Eutectic Solvents

**DOI:** 10.3390/ijms25126677

**Published:** 2024-06-18

**Authors:** Debora Procopio, Carlo Siciliano, Assunta Perri, Gabriela Guillena, Diego J. Ramón, Maria Luisa Di Gioia

**Affiliations:** 1 Department of Pharmacy, Health and Nutritional Sciences, University of Calabria, 87036 Rende, Italy; debora.procopio@unical.it (D.P.); carlo.siciliano@unical.it (C.S.); assunta.perri.ap@gmail.com (A.P.); 2 Department of Organic Chemistry, Institute of Organic Synthesis (ISO), University of Alicante, 03080 Alicante, Spain; gabriela.guillena@ua.es (G.G.); djramon@ua.es (D.J.R.)

**Keywords:** API synthesis, atenolol, epoxide ring-opening, green chemistry, green solvents

## Abstract

Atenolol, one of the top five best-selling drugs in the world today used to treat angina and hypertension, and to reduce the risk of death after a heart attack, faces challenges in current synthetic methods to address inefficiencies and environmental concerns. The traditional synthesis of this drug involves a process that generates a large amount of waste and other by-products that need disposal. This study presents a one-pot DES-based sustainable protocol for synthesizing atenolol. The use of the DES allowed the entire process to be conducted with no need for additional bases or catalysts, in short reaction times, under mild conditions, and avoiding chromatographic purification. The overall yield of atenolol was 95%. The scalability of the process to gram-scale production was successfully demonstrated, emphasizing its potential in industrial applications. Finally, the ‘greenness’ evaluation, performed using the First Pass CHEM21 Metrics Toolkit, highlighted the superiority in terms of the atom economy, the reaction mass efficiency, and the overall process mass intensity of the DES-based synthesis compared with the already existing methods.

## 1. Introduction

Racemic atenolol is currently among the top five best-selling drugs in the world and is manufactured under the names Tenormin^®^, Mylan^®^, and others [1]. First introduced in 1976 as a substitute for propranolol, atenolol is widely used in the treatment of hypertension, myocardial infarction, arrhythmias, and angina, as well as in conditions caused by reduced blood circulation and vasoconstriction, including migraine [2]. Atenolol belongs to the class of β-blockers and, as such, it is a selective β1 receptor antagonist [1,3]. It hinders the activity of substances present in the body, such as adrenaline, at the level of the heart and blood vessels, causing a consequent reduction in heart rate and blood pressure, as well as cardiac stress [4].

In 2022, the sales market for atenolol was more than USD 11 billion and it is estimated to reach USD 28 billion by the end of 2035. This growth is attributable to the worldwide increase in hypertension among people, along with the increased awareness of the risks associated with it [5].

**Scheme 1:**
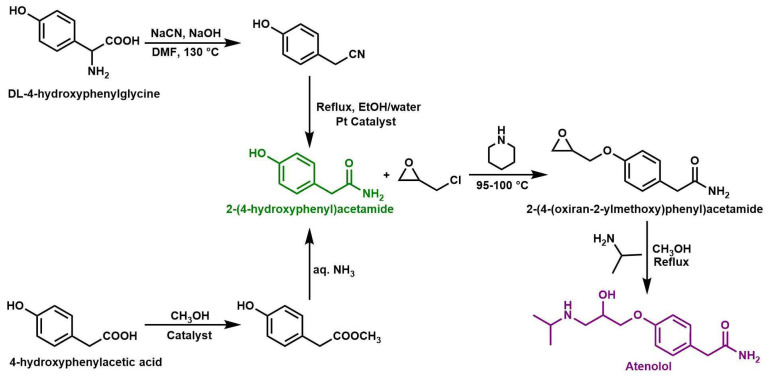
Industrial synthesis of atenolol [1,6,7]. A large excess of epichlorohydrin was used both as a reagent and as a solvent.

The initial patented chemical routes for the synthesis of atenolol used DL-4-hydroxyphenylglycine or 4-hydroxyphenylacetic acid as starting reagents to prepare 2-(4-hydroxyphenyl) acetamide (Scheme 1), which further reacted in subsequent steps to produce atenolol [6]. Currently, this industrial procedure is simplified by the commercial availability of 2-(4-hydroxyphenyl)acetamide, which reacts with a large excess of epichlorohydrin, acting both as a reagent and a solvent in the presence of piperidine to produce 2-(4-(oxiran-2-ylmethoxy)phenyl)acetamide. The latter compound further reacts with an excess of isopropylamine (IPA) to produce atenolol [1,6,7].

The industrial processes, however, suffer from severe drawbacks in terms of sustainability, as they generate substantial quantities of waste and other by-products that must be appropriately disposed of [8]. Over the years, a number of different strategies for the synthesis of atenolol and related compounds have been reported. Nevertheless, none of the proposed procedures can be considered green.

Most of these methods rely on the use of toxic or expensive unusual catalysts [9,10,11,12,13], Volatile Organic Compound (VOC)-based harmful solvents [9,10,11,12], as well as high reaction temperatures [14], harsh reaction conditions, long reaction times [15], and solubility issues [16], which can hamper the efficiency and scalability of the synthesis. Various strategies have been proposed for the enzymatic kinetic resolution of racemic atenolol, as well as for the enantioselective synthesis of the (*S*)-enantiomer to which the maximum β1-blocking activity is attributed [17,18,19,20,21]. Although the efficient methods that lead to the formation of enantiomerically pure (*S*)-atenolol can be of interest to the pharmaceutical industry and academia, the proposed routes use multistep procedures with a low overall yield, thus not aligning with the current environmental challenges [17,18,19]. A recent approach suggests the use of glycerol as an eco-friendly reaction medium for the last step in atenolol synthesis [22,23]. Consequently, the development of a more efficient and practical green route to the atenolol API remains an important synthetic goal to minimize the environmental impact of such pharmaceutical processes.

One of the most prominent environmental issues associated with pharmaceutical productions is the extensive use of solvents, including VOCs [24,25,26]. The downside of their widespread use is the fact that they represent the primary source of waste in the pharmaceutical industry. GlaxoSmithKline (GSK) reported that 80% of the waste in a complete life cycle analysis of the APIs is solvent-related [27,28,29]. In addition, most of them are hazardous solvents, and their high degree of toxicity and volatility not only leads to irreparable environmental risks but also to various adverse health effects [24,26]. Therefore, by limiting their use and opting for appropriate solvents, or the most environmentally friendly ones, significant reductions in the environmental impact of pharmaceutical production could be achieved [30,31].

In this regard, over the past decade, the European regulation concerning the ‘Registration, Evaluation, Authorisation and Restriction of Chemicals’ (REACH) introduced restrictions on the use of toxic solvents and, as a consequence, numerous pharmaceutical companies (Pfizer, AstraZeneca, Sanofi, GSK, etc.) have set up a Solvent Selection Guide for a careful “greener” replacement of dangerous solvents [26,32]. Recently, these guides were consolidated into the CHEM21 consortium, which was developed the CHEM21 guideline for solvent selection [33].

In this panorama, Deep Eutectic Solvents (DESs) have emerged as solvents for the 21st Century, marking a significant milestone in the pursuit of green and sustainable practices within the pharmaceutical industry [34,35,36,37,38,39,40,41].

These unique solvents, composed of natural compounds that form eutectic mixtures at relatively low temperatures, exhibit a wide range of appealing properties, such as low boiling points, low material costs, low toxicity, and biodegradability [35,42]. In addition, thanks to their tunable physicochemical attributes, they can be used for many applications, such as separation processes, extraction [26], and formulation [27,28], addressing the primary environmental concerns associated with VOCs [37,43,44,45]. DESs have shown a remarkable catalytic performance, emerging as a sustainable alternative to conventional solvents and catalysts in numerous chemical processes. These eutectic mixtures have proven effective in a range of applications, acting as both solvents and catalysts in organic synthesis and catalytic reactions. The ChCl-based DESs have been particularly successful in facilitating esterification, amidation, condensation, cross-coupling, cycloaddition, and oxidation reactions, with the added benefit of being reusable in both homogeneous and heterogeneous catalytic contexts [46,47,48,49].

Based on this background and our expertise in the use of the DESs as reaction media in organic synthesis [50,51,52,53,54], we aimed to unlock the potential of DESs to promote the green synthesis of APIs. Therefore, this work wants to address and reduce the environmental impact associated with atenolol synthesis using a DES as an eco-friendly reaction medium and streamlining the synthetic process.

## 2. Results and Discussion

### 2.1. DES-Based Atenolol Synthesis: Optimization of the First Step

**Scheme 2:**
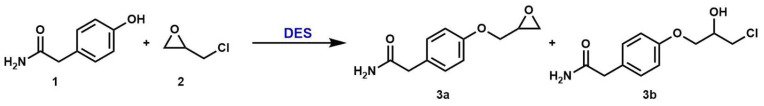
First step in the DES-based synthesis of atenolol.

Our study started by focusing on the first step of the industrial production of atenolol (Scheme 2) in DES.

Various authors have studied the ring opening of epoxides, employing choline chloride (ChCl)-based DESs as environmentally friendly and safe reaction media, affording the desired products favorable chemo-, regio-, and stereoselectivity [55,56,57]. Therefore, we chose to test some of the most successful DESs adopted in these transformations, composed using ChCl as a hydrogen bonding acceptor (HBA) and urea, glycerol, and ethylene glycol as hydrogen bonding donors (HBD).

The first attempted DES, known as “reline”, is a 1:2 molar mixture of naturally available choline chloride and urea. This DES is cost-efficient, biodegradable, non-toxic in the environment, and exhibits a negligible vapor pressure [58,59].

ChCl alone had no catalytic effect, being very scarcely soluble in epichlorohydrin within 8 h (entry 1, Table 1). Therefore, the optimization proceeded by suspending 2-(4-hydroxyphenyl) acetamide **1** in 1.0 g of ChCl:urea (1:2) DES and stirring the resulting mixture at 40 °C for 15 min. Epichlorohydrin (1 equiv.) was then added dropwise to the above-mentioned mixture and further stirred at room temperature (rt, 25 °C) without any additional solvents. After 15 h of reaction time, only a 54% conversion of **1** occurred (entry 2, Table 1). The evaporation of epichlorohydrin was accomplished. The final addition of water resulted in the formation of a precipitate that was analyzed using GC/MS, revealing the formation of a mixture of the glycidyl ether (**3a**) and halohydrin (**3b**) derivatives in a 80% and 20% yield, respectively. The conversion percentage did not significantly improve, even when the experiment was conducted at 40 °C, with extended reaction times and/or increased equivalents of epichlorohydrin used (entries 3 and 4, Table 1).

At this point, we wondered if the ChCl:glycerol (glyceline) and/or ChCl:ethylene glycol (ethaline) DESs that are widely acknowledged as new and effective catalysts for the ring opening of epoxides could perform better [55,56,57]. Notably, the effectiveness of the desired transformation increased when using ChCl:glycerol (Gly) (1:2) DES as the reaction medium, obtaining a good conversion of 2-(4-hydroxyphenyl)acetamide (**1**) with 90% selectivity towards **3a** (entry 5, Table 1). The conversion percentage of **1** reached 80% when the reaction was performed at 40 °C (entry 6, Table 1). Remarkably, the efficiency of this reaction improved upon switching to ChCl:ethylene glycol (EG) (1:2) as the reaction medium, due to a rapid and complete solubilization of the starting 2-(4-hydroxyphenyl)acetamide in this DES with respect to the others investigated. A very good selectivity towards **3a** was observed when the reaction was stirred at room temperature for 24 h. The resulting conversion reached 85% (entry 7, Table 1). The best conversion (99%) was achieved in only 6 h, when the reaction was performed at 40 °C (entry 8, Table 1). No improved result was obtained when carrying out the experiment using a 1:1 stoichiometric molar ratio between the starting compounds (entry 9, Table 1). Furthermore, ethylene glycol used alone (entry 10, Table 1) was ineffective even after 24 h. Thus, based on the observed results of the reaction performance, as well as from an energy-saving point of view, the following experiments were conducted under ChCl:EG DES. Of note, this reaction holds promise in terms of sustainability with respect to the traditional reaction reported in the literature, which required a higher temperature (95–100 °C), a large excess of epichlorohydrin, and use as a solvent and a reagent, in the presence of toxic piperidine as a catalyst.

### 2.2. Hypothesized DES-Catalyzed Reaction Mechanism

**Scheme 3:**
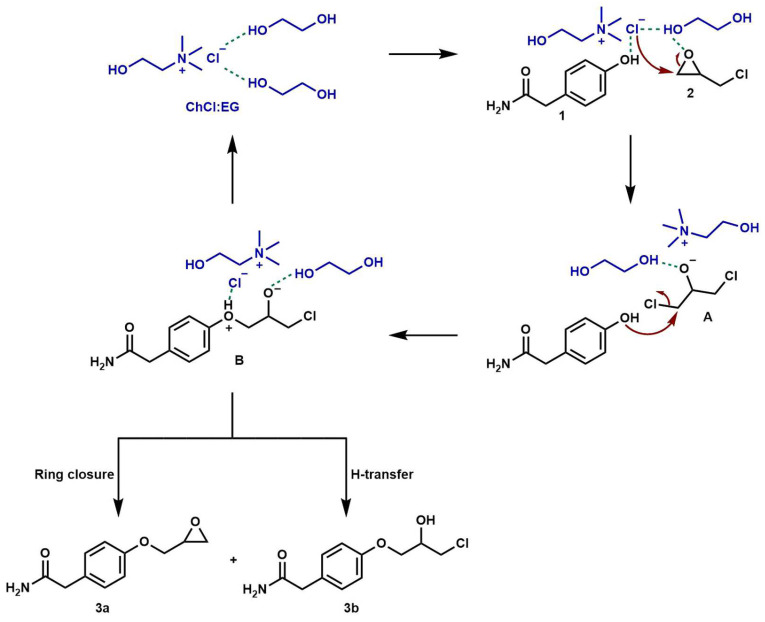
Hypothesized reaction mechanism for the DES-catalyzed formation of intermediates **3a** and **3b**.

Remarkably, the DES used went beyond the role of a solvent medium for the reaction, showing a catalytic effect too. Indeed, based on the data previously reported in the literature [55,60], we hypothesized a plausible mechanism for the reaction of epichlorohydrin with 2-(4-hydroxyphenyl)acetamide catalyzed using the ChCl:EG DES (Scheme 3).

**Figure 1:**
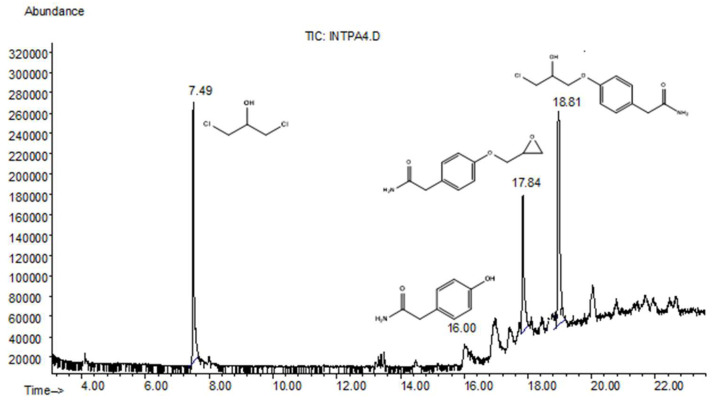
GC-MS analysis of the crude reaction mixture.

Initially, epichlorohydrin (**2**) is activated via the establishment of a hydrogen bonding between the ethylene glycol OH proton and the oxygen of the epoxide ring. This molecular arrangement enables the epoxide ring to open. Then, the HBA component of the DES assists with the epoxide ring opening. During this step, the chloride ion acts as a nucleophile and attacks the epoxide methylene term, forming the intermediate 1,3-dichloro-2-propanol (**A**). This hypothesis was confirmed by the results obtained using GC/MS analysis of an aliquot of the crude reaction mixture after 1 h reaction time (Figure 1).

In fact, the chromatogram showed a peak with a retention time of 7.49 min, and the mass spectrum confirmed the formation of 1,3-dichloro-2-propanol (see Supplementary Materials). Successively, the nucleophilic attack of the phenol function of 2-(4-hydroxyphenyl)acetamide (**1**) on 1,3-dichloro-2-propanol (A) generates another intermediate (B), which in turn cyclizes to produce the glycidyl ether product **3a**, through the chloride elimination. The non-quantitative yields in **3a** were attributed to the formation of a secondary product, the chlorohydrin open-chain **3b**, through a proton transfer (Scheme 3).

A notable benefit of conducting the reaction in the DES was the direct precipitation of compounds **3a** and **3b** as a mixture, by adding water to the reaction mixture. Water competes for interactions with DES anions, disrupting their ability to dissolve the intermediates and leading to their precipitation from DES–water mixtures [51]. This process eliminates the need for laborious work-up procedures or column chromatographic purification.

**Figure 2:**
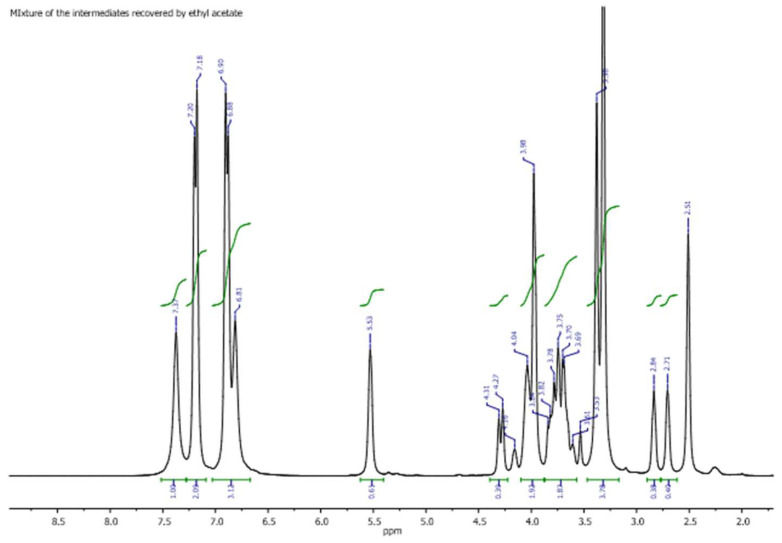
The ^1^H-NMR spectrum of an aliquot of the crude product from the optimized reaction (entry 8, Table 1) was obtained. The signal at 2.51 ppm corresponds to the solvent used for the NMR analysis and serves as the reference for the spectrum calibration.

In order to provide further additional support for the proposed mechanism, we conducted an in-depth high-resolution NMR analysis, adopting advanced mono- and two-dimensional homonuclear techniques (Figure 2). The objective was to elucidate the structure of the key intermediate in the synthesis process of atenolol.

The ^1^H NMR spectrum displayed clearly distinguishable and resolved signals that correlate with the oxyranic structure (**3a**) alone. All the spectral windows highlight the signals of the proton resonances of each of the proton spin systems of the molecular structure, together with a set of signals not generated by the protons of the oxyranic intermediate, but which can be easily attributed to residues of DES present in the sample and deriving from the extraction with the organic solvent. Starting from the spectral window of the shielded proton resonances, the pair of signals at 2.71 and 2.84 ppm attributable to the methylene diastereotopic protons of the oxyranic ring is evident. The remaining proton of the three-membered heterocyclic ring generates a fairly deshielded resonance signal centered at 4.29 ppm; the same spectral window contains the signals attributable to the diastereotopic protons of the oxygen-bound methylene group of the aromatic ring. Aromatic protons generate resonances with signals that are detectable in the spectrum in the form of a pair of apparent doublets at 6.89 and 7.19 ppm. The pair of methylene protons in the α position to the amide carbonyl resonate in the form of a singlet centered at 3.98 ppm, while the two chemically non-equivalent protons of the NH_2_ group provide the two signals at 6.81 and 7.37 ppm.

**Figure 3:**
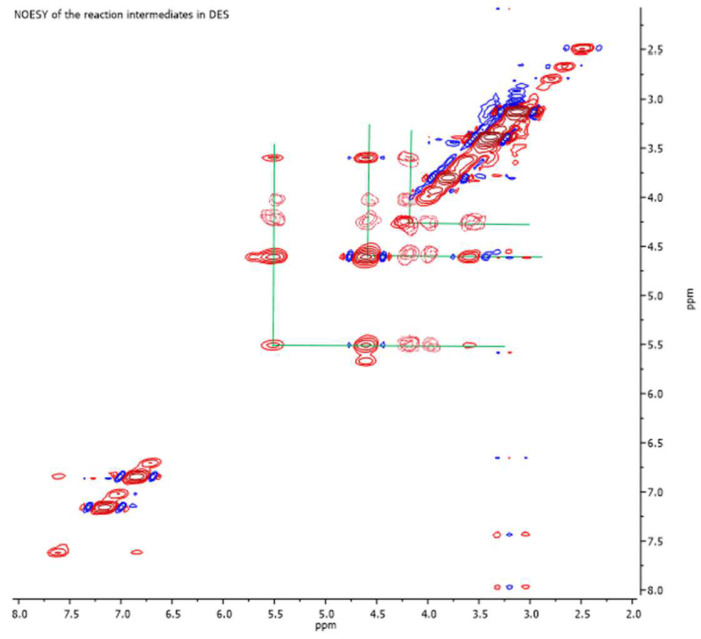
The ^1^H,^1^H-2D-Homonuclear NOESY spectrum recorded on a sample containing the oxyranic intermediate, in the presence of the ChCl:EG DES.

To attribute the remaining resonance signals that were not generated by the protons of the molecular structure of **3a** and hypothesized to be due to the protons of the pair of components of DES, a proton spectrum of an aliquot of the freshly prepared DES was recorded (see the Supplementary Materials). Finally, the absence of signals attributable to the hypothetical structure of a chlorohydrin open-chain intermediate (**3b**), at least within the limits of the sensitivity of the NMR instrumental techniques used, would confirm the formation of oxirane as the only one key intermediate during the process under consideration. The chemical shift value of the resonance signal generated using the methine proton resonance of the oxyranic ring can corroborate this hypothesis. In fact, this value (4.29 ppm) is not common for structurally simple oxiranes, as it is significantly downfield shifted due to the transfer of electron density to the oxygen atom, which is supposed to be involved in the hydrogen bond networks with the OH group of ethylene glycol. On the other hand, this conclusion is in total agreement with the results obtained using GC-MS analysis performed on the same raw sample subjected to the spectroscopic investigation. To further confirm the hypothesis, a two-dimensional homonuclear NOESY correlation spectrum was recorded on the same sample for which the spectrum of the oxyranic intermediate was acquired in the presence of the DES (Figure 3).

The plot of the two-dimensional homonuclear spectrum clearly highlights the spatial correlations between the choline OH proton (5.50 ppm), the ethylene glycol OH (4.52 and 4.04 ppm), the oxirane CH proton (4.19 ppm), and the glycol and choline methylene systems (range between 3.50 and 3.80 ppm), confirming the installation of a network of hydrogen bonds whose main partners are the oxyranic ring (via its oxygen atom) and the ethylene glycol. The same downfield shift due to the OH-O interaction would also be operative on the methylene proton pair of the three-terminus ring, thus determining the activation of the CH_2_ group at the nucleophilic attack that completes the synthesis of atenolol.

### 2.3. DES-Based One-Pot Two-Step Synthesis of Atenolol API

**Scheme 4:**
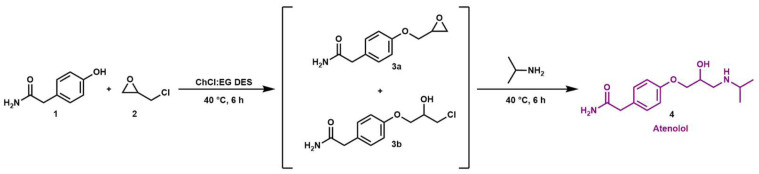
DES-based synthesis of atenolol.

With these data in hand, and considering that the final step of the industrial production of atenolol API (see Scheme 4) also involves a ring-opening reaction, we envisaged the feasibility of performing a one-pot, two-step synthesis of atenolol in the ChCl:EG DES. A tool to reduce waste during chemical reactions is the one-pot synthesis approach that avoids the purification and isolation of the intermediates, thereby reducing the amount of the solvents used [61]. As a result, one-pot synthesis holds promise for addressing the various challenges encountered in pharmaceutical and synthetic laboratories, including reduced exposure time and simplified procedures.

Thus, we replicated the experiment involving the reaction between 2-(4-hydroxyphenyl)acetamide **1** and epichlorohydrin **2** in the DES to yield the corresponding glycidyl ether **3a** and chlorohydrin **3b** intermediates. The excess of epichlorohydrin was removed under a vacuum, and the IPA (3 equiv.) was directly added to the reaction mixture, which was further stirred at 40 °C (Scheme 4).

As expected, the reaction went to completion in 6 h, and then the excess IPA was removed via a vacuum evaporation. The addition of water resulted in the precipitation of a white solid, which was subsequently washed with water and dried to produce the corresponding atenolol (**4**) with an overall yield of 95%. The characterization of atenolol was performed using NMR spectroscopy.

As already mentioned, the ring opening of the epoxide **3a** by isopropylamine was conceivably aided by the hydrogen bond network formed by the components of the DES. Consequently, isopropylamine attacked the less-hindered side of the epoxide, yielding the desired atenolol (**4**). Atenolol was successfully isolated and recovered without the need for chromatographic purification.

### 2.4. Scaling Up the Synthesis of Atenolol in ChCl:EG DES

Given the estimated growth of the atenolol market [5] and its relevance as an antihypertensive drug, the potential applicability for large-scale manufacturing of the developed method has been evaluated. In order to prove the applicability of the method, we also carried out the synthesis of **4** on a 1 g scale and a 10 g scale. In this regard, the entire process was scaled up to 1.0 g of 2-(4-hydroxyphenyl)acetamide (**1**) (6.62 mmol) using 3 mL of the ChCl:EG DES. Following the detailed procedure outlined above, atenolol was obtained with a yield of 95%. Further scaling up the reaction to 10.0 g of **1** (66.2 mmol) in 30 mL of the DES resulted in atenolol being recovered with an excellent yield, indicating the potential switch to industrial-scale production using the developed method.

### 2.5. Recovery and Recycling of DES

From an industrial perspective, the recovery and reuse of the DES play a significant role in addressing both economic and environmental concerns within chemical processes [62,63,64]. The assessment of DES reusability is detailed in Table 1 (entry 8). After a complete conversion of the precursors, water was added to the reaction mixture, and the solid product was filtered off. The ChCl:EG DES was then recovered from the filtrate through evaporation of the water phase under a vacuum at 80 °C, allowing for its reuse with a minimal loss of activity.

### 2.6. Green Metrics Evaluation

At this point, we aimed to provide a comprehensive understanding of the environmental impact of our synthetic approach, thereby contributing to the advancement of sustainable manufacturing practices in the pharmaceutical industry. In line with the first principle of Green Chemistry, we evaluated the E-factor of the proposed procedure. The E-factor is a green metric that estimates the kilograms of waste generated per kilogram of the product achieved and stands as one of the pioneer metrics for assessing the sustainability of a process. The optimal E-factor value is 0 [63].

In this regard, the green advance of our work is the lack of purification of the product using chromatography and the reduction of the waste generated compared with the traditional synthesis. Specifically, we found an E-factor value of 3 for the first step. The cumulative E factor extends the concept of the E factor to consider the entire synthetic route, including all the intermediate steps. A cumulative E-factor value of 22 was calculated for this process. Moving beyond the use of the E-factor, a deep insight evaluation of the greenness of our method using the First Pass CHEM21 Metrics Toolkit [33,65] was performed, enabling the comparison of these values (whenever possible) with the conventional industrial routes of producing atenolol [66,67] in order to ensure a truly holistic approach. Table 2 details the values calculated for the main green metrics, including the atom economy (AE), the reaction mass efficiency (RME), the optimum efficiency (OE), the mass intensity (MI), and the process mass intensity (PMI) metrics, with a breakdown of the latter for “chemicals” (reactants, reagents, and catalyst) (PMI_RRC_), and chemical and reaction solvents (PMI_Solv_).

Most of the parameters under consideration proved the greenness of our process among the others. Specifically, the RME value of this methodology was 54%, in contrast to 6% and 14%, which were obtained for the other procedures. Consequently, the OE value (61.6%), calculated as the ratio of the RME and the AE, resulted in a better percentage than the others (7.1 and 15.5%, respectively). Additionally, a notable difference in the case of the PMI values was observed. The PMI was defined as the total mass of the reactants used to produce a specified mass of product, including the solvents, work-up and purification steps, additives, and catalysts. It represents the most important metric for pharmaceutical companies in evaluating the greenness of a chemical process [68]. On this matter, an ideal process should have a PMI value close to 0; higher values of the PMI mean a greater negative environmental impact of the method. In our case, the PMI, PMI_RRC_, and PMI_Solv_ values were 3.9, 1.8, and 2.0, respectively. The same parameters were also evaluated for the patented routes that were taken into account, resulting in higher values. Therefore, from a green chemistry perspective, it is clear that the DES-based one-pot synthesis of atenolol offered the best route with respect to the classical procedures. Likewise, the overall qualitative evaluation further highlighted the greenness of the developed method. The qualitative parameters played a pivotal role, particularly in their assessment of the intrinsic safety of the utilized materials and the efficiency of energy. These parameters were systematically classified into three categories: green, amber, and red flags, indicating the desirability of the preferred conditions, acceptability with certain concerns, and the avoidance of undesirable situations, respectively [65]. More specifically, the flags assigned to the reagents and work-up tabs aligned with the results obtained in the mass metrics. Both the patented procedures raised concerns, marked by a red flag regarding the solvents and catalyst/enzyme usage. Additionally, concerning the energy consumption, an amber flag was noted for Jang et al.’s U.S. Patent 005290958A [66], while Kitaori et al. [67] received a green flag. Nevertheless, in an overall view, our approach outperformed in terms of all the parameters considered, making this approach more desirable.

## 3. Materials and Methods

### 3.1. General Remarks

The starting materials were purchased from Aldrich (Merck, Rahway, NJ, USA) and used as received without further purification, unless otherwise specified. The reactions were monitored using thin-layer chromatography (TLC) using Schleicher & Schuell F1400/LS 254 plates (BioScience GmbH, Dassel, Germany) coated with a 0.2 mm thick layer of silica gel and using GC/MS analysis. The mass spectra (EI) were acquired using a Shimadzu QP-5000 mass spectrometer (Shimadzu, Columbia, MD, USA) at 70 eV, providing fragment ions at *m*/*z* with relative intensities (%). The ^1^H NMR spectra were recorded at 300 MHz on Bruker AC-300 NMR spectrometers (Bruker, Billerica, MA, USA) in a proton-coupled mode. The ^13^C NMR spectra were recorded at 75.5 MHz on a Bruker AC-300 NMR spectrometer (Bruker, Billerica, MA, USA) in a proton decoupling mode at 20 °C. The chemical shifts were expressed in δ (parts per million) and coupling constants (*J*) in Hertz.

### 3.2. Experimental Procedure for the Preparation of DES

The DESs used were prepared following literature-reported procedures, using the heating and stirring method [69]. Specifically, all DESs were prepared by employing choline chloride as the hydrogen bond acceptor (HBA) and urea, glycerol, and ethylene glycol as hydrogen bond donors (HBD) in a 1:2 molar ratio, respectively. The components were placed in a heated glass flask at approximately 60 °C and continuously stirred using a heating plate equipped with a magnetic stirrer, for 2 h. The obtained DESs were used without any further purification.

### 3.3. Experimental Procedure for the One-Pot, Two-Step Synthesis of Atenolol

In a 25 mL round-bottom flask containing 0.6 mL of the ChCl:EG DES, 2-(4-hydroxyphenyl)acetamide (200 mg, 1 equiv.) was introduced, and the mixture was stirred magnetically at 40 °C. Following the immediate solubilization of the amide, epichlorohydrin (0.184 g, 1.5 equiv.) was added dropwise, and the reaction mixture was further stirred at a temperature of 40 °C for 6 h. The reaction progress was monitored using TLC and GC/MS. Upon completion, the unreacted epichlorohydrin was removed using evaporation under a reduced pressure. After removing the excess epichlorohydrin, the reaction mixture was further subjected to magnetic stirring and heated to a temperature of 40 °C. Subsequently, isopropylamine (0.235 g, 3 equiv.) was added dropwise. The reaction progress was monitored using TLC and GC/MS. Upon completion after 6 h, the excess isopropylamine was removed using evaporation under reduced pressure. Afterwards, water was added to the reaction mixture so that the hydrogen bonds between the DES components might break, causing the precipitation of atenolol (**4**) as a white solid (0.33 g, 95% yield). The characterization of atenolol was performed using NMR and GC-MS analysis (purity, no less than 98%, using GC-MS and 1H qNMR).

## 4. Conclusions

The developed method allows for an environmentally friendly and economically viable one-pot, two-step synthesis of atenolol in the ChCl:EG DES, demonstrating the versatility of the system. The proposed mechanism suggests the involvement of a hydrogen bonding network between the DES components and the reagents, assisting the epoxide ring opening and thus the formation of atenolol. The scalability of the process to gram-scale production was successfully demonstrated, emphasizing its potential in industrial applications. Furthermore, the recovery and reuse of the catalysts/DES system for the first step were explored, showing a minimal loss of activity after several cycles. The ‘greenness’ evaluation using the First Pass CHEM21 Metrics Toolkit highlighted the superiority of the DES-based synthesis compared with the already existing methods.

## Figures and Tables

**Table 1:** Optimization data for the preparation of intermediates of atenolol.

Entry	DES	T (°C)	t (h)	Conversion ^c^ (%)	Selectivity ^c^	
					3a	3b
entry 1 ^a^	ChCl	rt	8	No conversion	-	-
entry 2 ^a^	ChCl:U (1:2)	rt	15	54	80	20
entry 3 ^b^	ChCl:U (1:2)	rt	24	61	80	20
entry 4 ^b^	ChCl:U (1:2)	40	24	68	85	15
entry 5 ^b^	ChCl:gly (1:2)	rt	24	73	90	10
entry 6 ^b^	ChCl:gly (1:2)	40	24	80	90	10
entry 7 ^b^	ChCl:EG (1:2)	rt	24	85	95	5
**entry 8 ^b^**	**ChCl:EG (1:2)**	**40**	**6**	**>99 ^d^**	**95**	**5**
entry 9 ^a^	ChCl:EG (1:2)	40	6	65	93	7
entry 10 ^b^	EG	40	24	No conversion	-	-

^a^ 2-(4-hydroxyphenyl)acetamide and epichlorohydrin were used in a molar stoichiometric ratio of 1:1; ^b^ 2-(4-hydroxyphenyl)acetamide and epichlorohydrin were used in a molar stoichiometric ratio of 1:1.5; ^c^ Conversion and selectivity were assessed using GC-MS analysis; ^d^ The DES was recovered and successfully reused for up to three cycles. No changes in yields were observed.

**Table 2:** Greenness evaluation of the classical [66,67] and DES-based synthesis of atenolol using the CHEM21 Metrics Toolkit.

Entry	Ideal Value	Our Work	Jang et al. [66]	Kitaori et al. [67]
AE (%)	100	88	88	88
RME (%)	100	54	6	14
OE (%)	100	61.6	7.1	15.5
PMI	1	3.9	25.2	10.8
PMI_RRC_	1	1.8	16.0	7.5
PMI_Solv_	1	2	9.2	3.3
Solvents (First Pass)	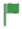	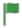	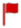	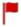
Catalyst/enzyme (First Pass)	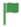	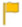	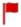	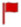
Energy (First Pass)	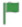	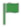	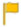	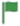
Work-up	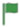	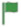	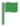	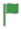

## Data Availability

All the relevant supporting data can be found within the manuscript or the accompanying Supplementary Files. Separate files are available for the CHEM21 Metrics Toolkit.

## Supplementary Materials

The following supporting information can be downloaded at: https://www.mdpi.com/article/10.3390/ijms25126677/s1, Scale-up procedure; Characterization data; Spectral data. Ref. [70] is cited in the Supplementary Materials file.
