# Supplementary material for: Sustainable Synthesis of the Active Pharmaceutical Ingredient Atenolol in Deep Eutectic Solvents
Source: Int J Mol Sci. 2024 Jun 18;25(12):6677. doi: 10.3390/ijms25126677 (PMC11203897; doi:10.3390/ijms25126677)
Supplement: Supplementary file 1 [file ijms-25-06677-s001.zip › ijms-3025743-supplementary.pdf]

Sustainable Synthesis of the Active Pharmaceutical Ingredient Atenolol in Deep Eutectic Solvents

Debora Procopio,[1,#] Carlo Siciliano,[1,#] Assunta Perri,[1] Gabriela Guillena,[2] Diego J. Ramón[2] and Maria Luisa Di Gioia,[1,*]

1. General remarks

The starting materials were purchased from Aldrich (Merck) and used as received without further purification unless otherwise specified. The reactions were monitored by thin-layer chromatography (TLC) using Schleicher & Schuell F1400/LS 254 plates coated with a 0.2 mm thick layer of silica gel and by GC/MS analysis. Mass spectra (EI) were acquired on a Shimadzu QP-5000 mass spectrometer at 70 eV, providing fragment ions at m/z with relative intensities (%). ^{1}H NMR spectra were recorded at 300 MHz on a Bruker Avance 300 NMR spectrometer, equipped with a 5 mm tube BB0 probe, and an automated temperature control unit. ^{13}C NMR spectra were recorded at 75 MHz in a total proton-decoupled mode. ^{1}H and ^{13}C one-dimensional spectra were recorded with a TD of 128k, and zero-filled with a SI of 256k. Homonuclear 2D-NOESY spectra were acquired by applying the native instrumental Bruker pulse sequence, using the default parameters. All one- and two-dimensional spectra were recorded at 298 K, using DMSO-d_6 as the solvent, with a 100% isotopic purity. Aliquots of 35 mg of each sample and standards were solubilized in 0.6 mL of deuterated solvent and subjected to spectroscopic analysis. Chemical shifts were expressed in ppm and referred to the residual frequency of the deuterated solvent (2.51 and 40 ppm for ^{1}H and ^{13}C spectra, respectively). Coupling constants (*J*) are expressed in Hertz. Atenolol purity grade was determined by qNMR absolute quantification method [1] (calibrant $CHCl_3$, analytical reagent purity grade >99,8%)

2. General procedure for the preparation of the DES

The DESs used were prepared following literature-reported procedures, using the heating and stirring method. Specifically, all DESs were prepared by employing choline chloride as the hydrogen bond acceptor (HBA) and urea, glycerol, or ethylene glycol as hydrogen bond donors (HBD) in a 1:2 molar ratio, respectively. The components were placed in a round bottom flask at approximately 60°C and continuously stirred using a heating plate, equipped with a magnetic stirrer, for 2 hours. The obtained DESs were used without any further purification.

[1] Pauli, G.F.; Chen, S.N.; Simmler, C.; Lankin, D.C.; Gödecke, T.; Jaki, B.U.; Friesen, J.B.; McAlpine, J.B.; Napolitano, J.G. Importance of Purity Evaluation and the Potential of Quantitative 1H NMR as a Purity Assay *J. Med. Chem.* **2014**, *57*, 22, 9220–9231

3. Experimental procedure for the one-pot, two steps synthesis of atenolol

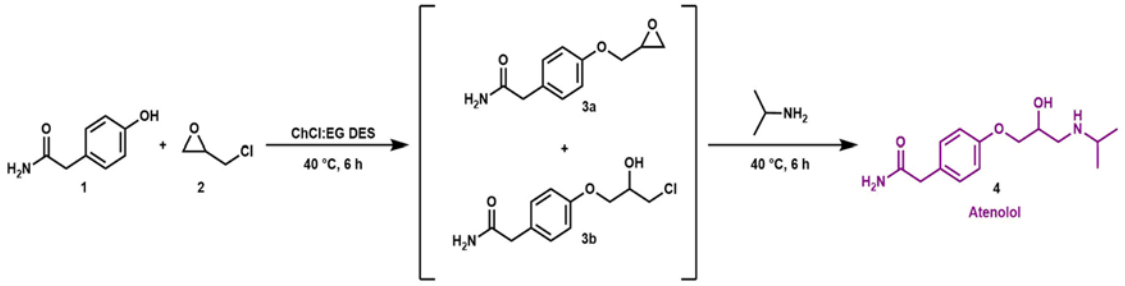

In a 25 mL round-bottom flask containing 0.6 mL of ChCl:EG DES, 2-(4-hydroxyphenyl)acetamide (200 mg, 1 equiv.) was added, and the mixture was stirred magnetically at 40 °C. Then, epichlorohydrin (0.184 g, 1.5 equiv.) was added dropwise, and the reaction mixture was kept at 40 °C for 6 hours. The reaction progress was monitored by TLC and GC/MS. Upon completion, the excess epichlorohydrin was removed by evaporation under reduced pressure. Subsequently, isopropylamine (0.235g, 3 equiv.) was added dropwise and the reaction mixture was stirred at 40 °C. Upon completion of the reaction (after 6 hours), the excess isopropylamine was removed by evaporation under reduced pressure. Afterwards, water (5 mL) was added to the reaction mixture so that the hydrogen bonds between the DES components might break causing the precipitation of atenolol (**4**) as a white solid (0.33 g, 95% yield). The obtained atenolol was characterized by ^{1}H-NMR and ^{13}C NMR analysis.

4. Scaling-up the one-pot, two steps synthesis of atenolol (1 g)

The reaction was carried out on a 1 g scale of 2-(4-hydroxyphenyl)acetamide (**1**) using 3 mL of ChCl:EG DES: in a 25 mL round-bottom flask containing 3 mL of ChCl:EG DES, 2-(4-hydroxyphenyl)acetamide (1.0 g, 1 equiv.) was added, and the mixture was stirred magnetically at 40 °C. Epichlorohydrin (0.918 g, 1.5 equiv.) was then added dropwise, and the reaction mixture was further stirred for 6 hours at a temperature of 40 °C. The reaction progress was monitored by TLC and GC/MS. Upon completion, the excess epichlorohydrin was removed by evaporation under reduced pressure. Subsequently, isopropylamine (1.173 g, 3 equiv.) was added dropwise to the reaction mixture and magnetically stirred at 40 °C and monitored via TLC

and GC/MS. Upon completion of the reaction (after 6 hours), the excess isopropylamine was removed by evaporation under reduced pressure. Atenolol recovery was performed by adding an aqueous solution of $NaHCO_3$ (10 mL), followed by extraction with ethyl acetate (5 mL x 3). Next, the organic phase was dehydrated with Na_2SO_4, filtered, and then subjected to evaporation under reduced pressure. The obtained solid was washed with a mixture of hexane (5 mL) and cyclopentyl methyl ether (1 mL), further with hexane (5 mL x 2), and finally subjected to evaporation under reduced pressure. Atenolol was afforded in 95% yield (1.67 g).

5. Scaling up the one-pot, two steps synthesis of atenolol (10 g) :

The reaction was carried out on a 10 g scale of *p*-hydroxyphenylacetamide using 30 mL of ChCl:EG DES: In a 100 mL round-bottom flask containing 30 mL of ChCl:EG DES, *p*-hydroxyphenylacetamide 10 g, 1 equiv.) was added, and the mixture was stirred magnetically at 40 °C. Epichlorohydrin (9.181 g, 1.5 equiv.) was then added dropwise, and the reaction mixture was further stirred for 6 hours at a temperature of 40 °C. The reaction progress was monitored by TLC and GC/MS. Upon completion, the excess epichlorohydrin was removed by evaporation under reduced pressure. Subsequently, isopropylamine (11.731 g, 3equiv.) was added dropwise to the reaction mixture and magnetically stirred at 50 °C and monitored via TLC and GC/MS. Upon completion of the reaction (after 6 hours), the excess isopropylamine was removed by evaporation under reduced pressure. Atenolol recovery was performed by adding an aqueous solution of $NaHCO_3$ (15 mL), followed by extraction with ethyl acetate (15 mL x 3). Next, the organic phase was dehydrated with Na_2SO_4, filtered, and then subjected to evaporation under reduced pressure. The obtained solid was washed with a mixture of hexane (15 mL) and cyclopentyl methyl ether (3 mL), further with hexane (15 mL x 2), and finally subjected to evaporation under reduced pressure. Atenolol was afforded in 95% yield (16.74 g).

6. Characterization data

2-(4-(2-oxiran-2-ylmethoxy)phenyl)benzeneacetamide (3a):

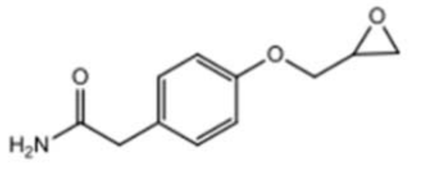

White solid. ^{1}H NMR (300MHz, DMSO-d6) 7.37 (br s, 1H, NH), 7.19 (d, 2H, ArH), 6.89 (d, 2H, ArH), 6.81 (s, 1H, NH), 4.29 (m, 1H, CH oxyranic), 3.98 (s, 2H, CH_2CONH_2), 3.82- 3.69 (m, 2H, OCH_2), 2.84 (s, 1H, CH_2 oxyranic diast), 2.71 (s, 1H, CH_2 oxyranic diast) ppm. EI MS (m/z, %): 207 (43, $M^{+\cdot}$), 191 (2), 163 (100), 133 (10), 107 (99), 89 (12), 77 (20), 57 (26), 44 (22)

2-(4-(3-chloro-2-hydroxypropoxy)phenyl)acetamide (3b):

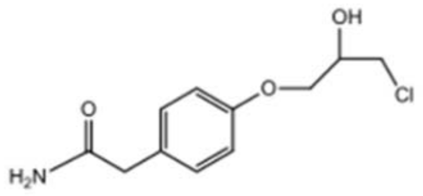

EI MS (m/z, %): 243/245 ($M^{+\cdot}$, 20/7%), 199/201 (49/17), 131 (9), 107 (100), 89 (10), 77 (13), 57 (6), 44 (12).

Atenolol (4):

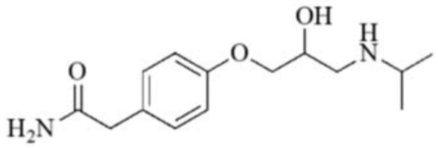

White solid 95%. ^{1}H NMR (300 MHz, DMSO-d_6) δ 7.39 (s, 1H, NH_2), 7.16 (m, 2H, ArH), 6.86 (d, 3H, ArH, NH_2), 4.97 (br s, 1H, OH), 3.95-3.83 (m, 3H, CHOH, CH_2CHOH), 3.29 (s, 2H, CH_2CONH_2), 2.76-2.66 (m, 2H, CH_2NH), 2.57 (m, 1H, $CH(CH_3)_2$), 1.51 (br s, 1H, NH), 0.98-95 (m, 6H, $CH(CH_3)_2$) ppm; ^{13}C NMR (75 MHz, DMSO-d_6) δ 173.6, 158.0, 130.7, 129.8, 114.9, 80.0, 68.5, 50.7, 48.9, 42.1, 23.4, 22.6 ppm. EI MS (m/z, %): 251 (1), 222 (3), 190 (1), 116 (1), 107 (5), 100 (1), 79 (1), 72 (100), 56 (4), 44 (4).

7. Spectral Data

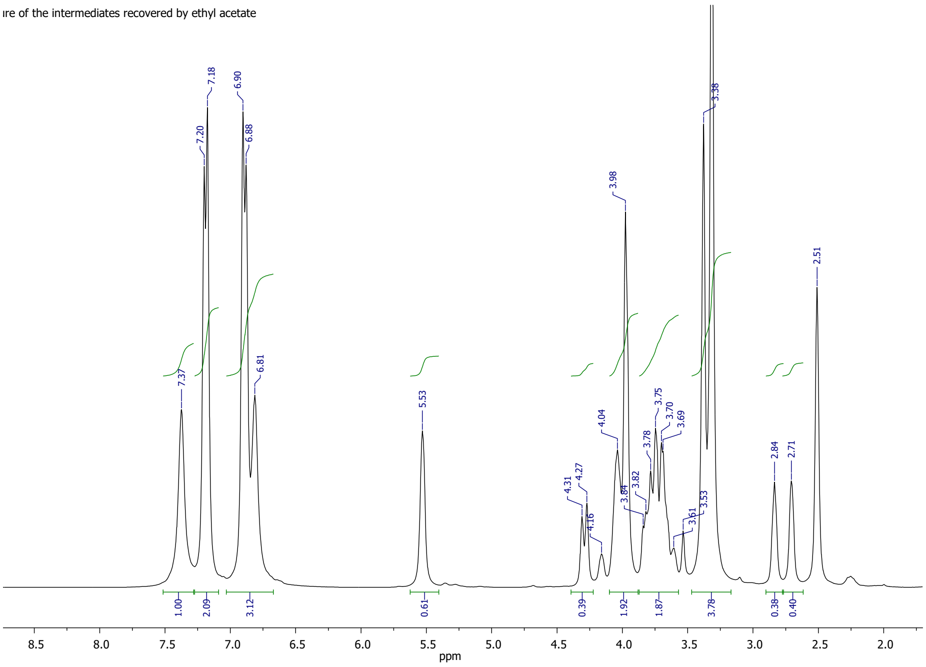

Figure SI 1. HR-^{1}H-NMR spectrum of an aliquot of a crude mixture of the two intermediates **3a** and **3b**.

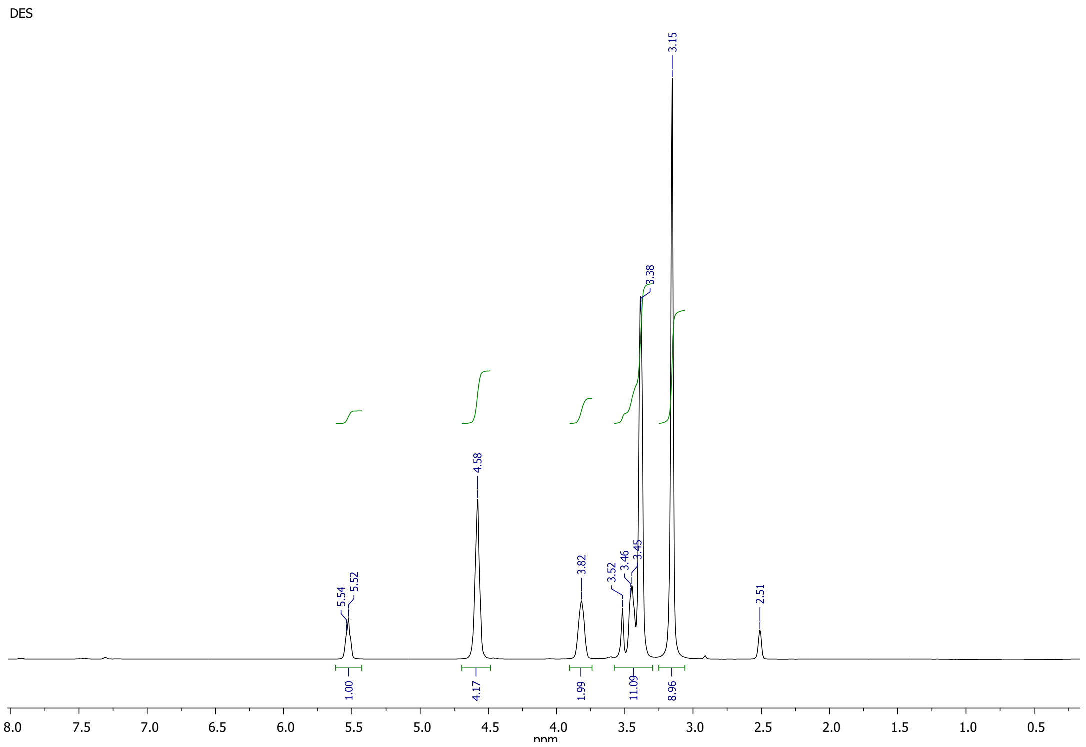

Figure SI 2. ^{1}H-HR-NMR spectrum recorded on an aliquot of freshly prepared ChCl:EG DES.

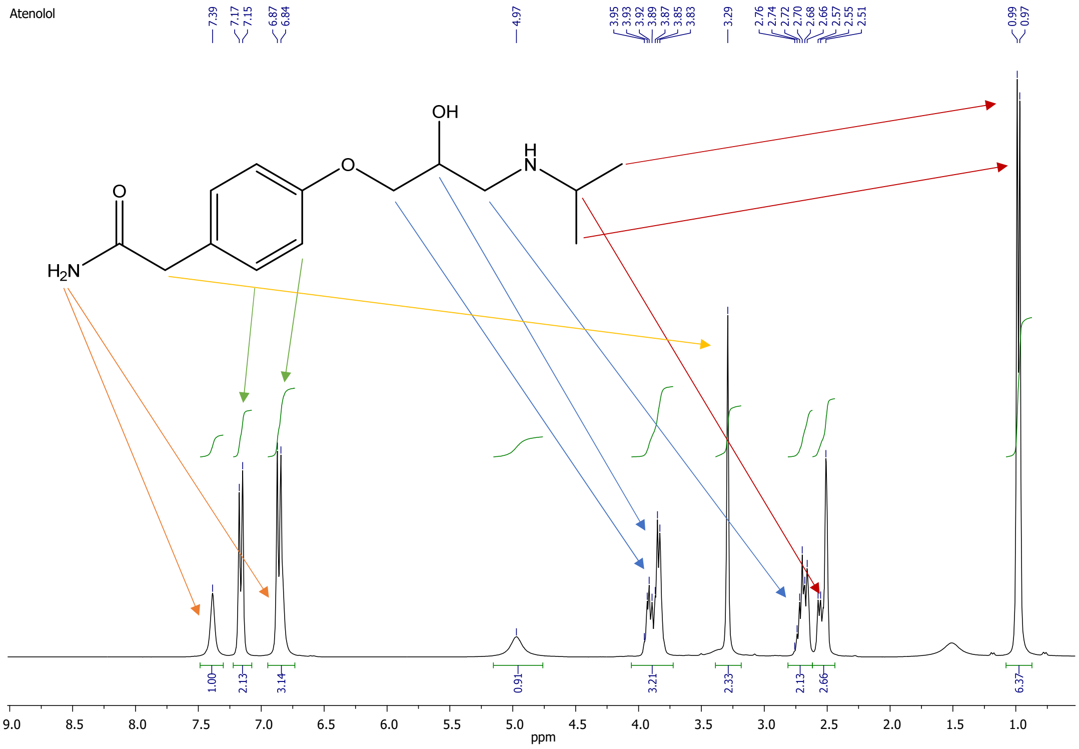

Figure SI 3. ^{1}H-HR-NMR spectrum of Atenolol.

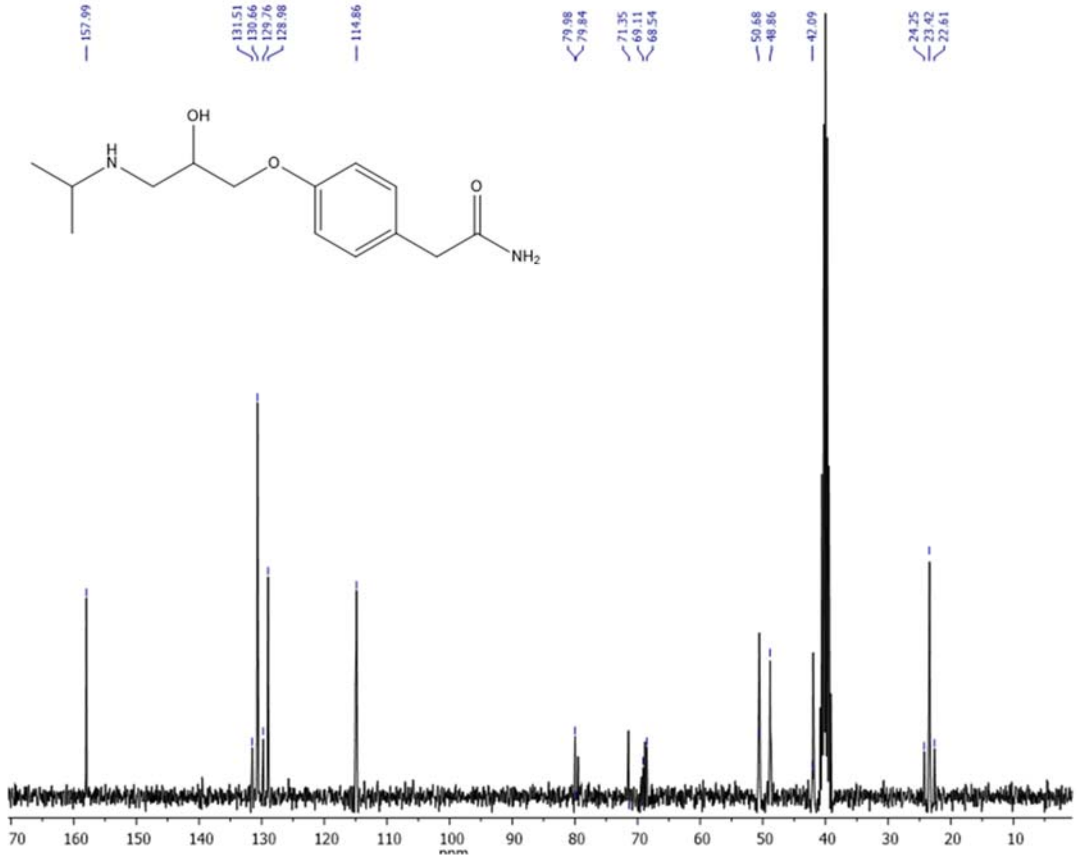

Figure SI 4. ^{13}C-HR-NMR spectrum of Atenolol.

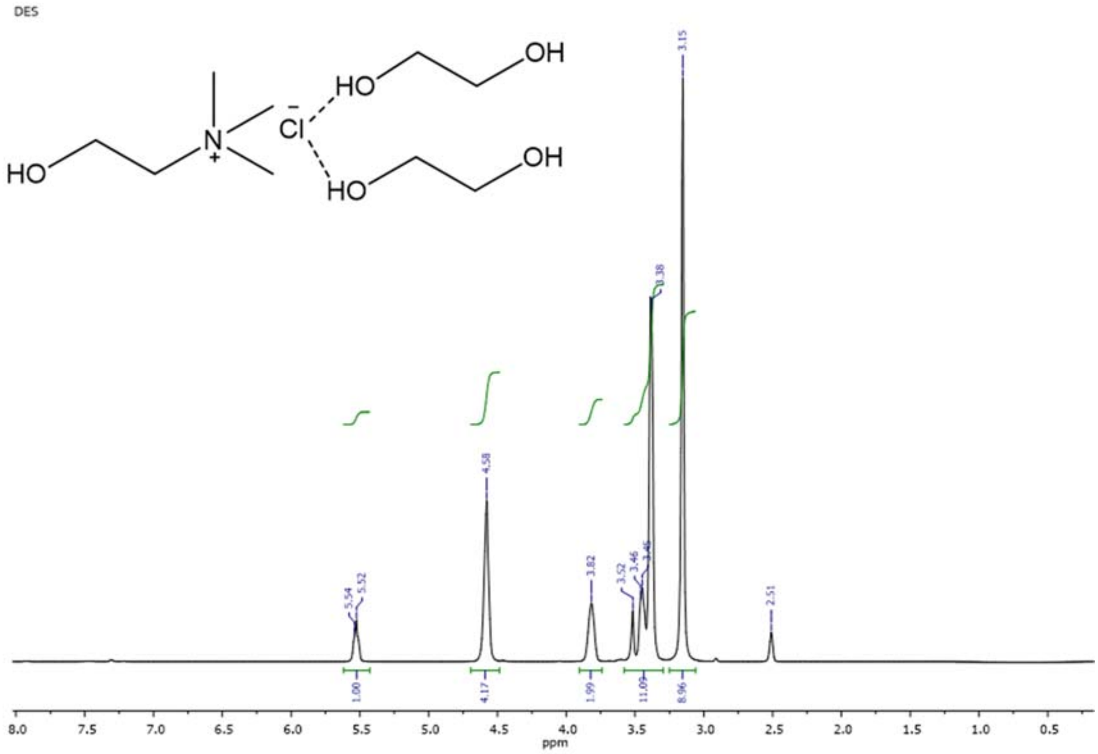

Figure SI 5. ^{1}H NMR of an aliquot of DES in DMSO-d6

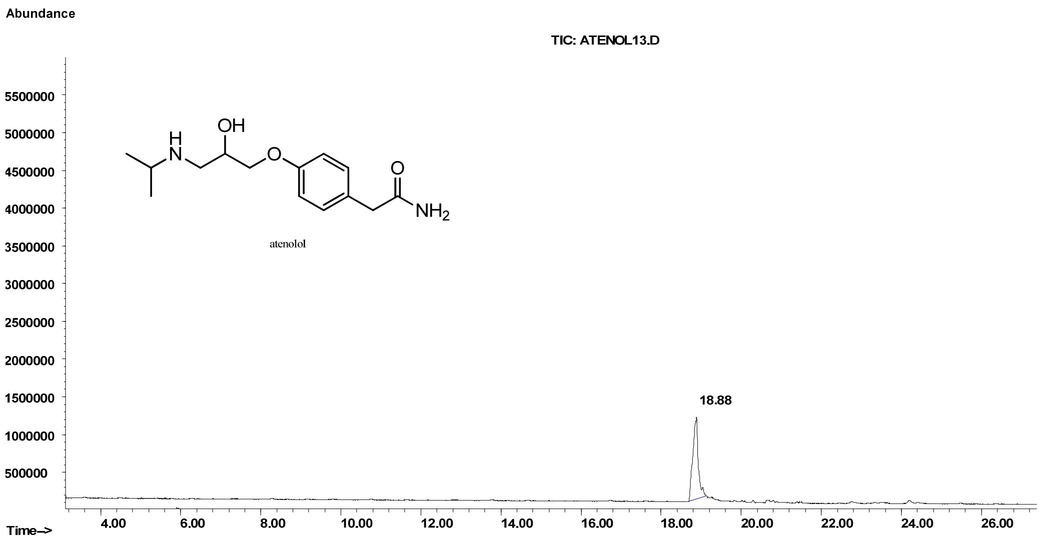

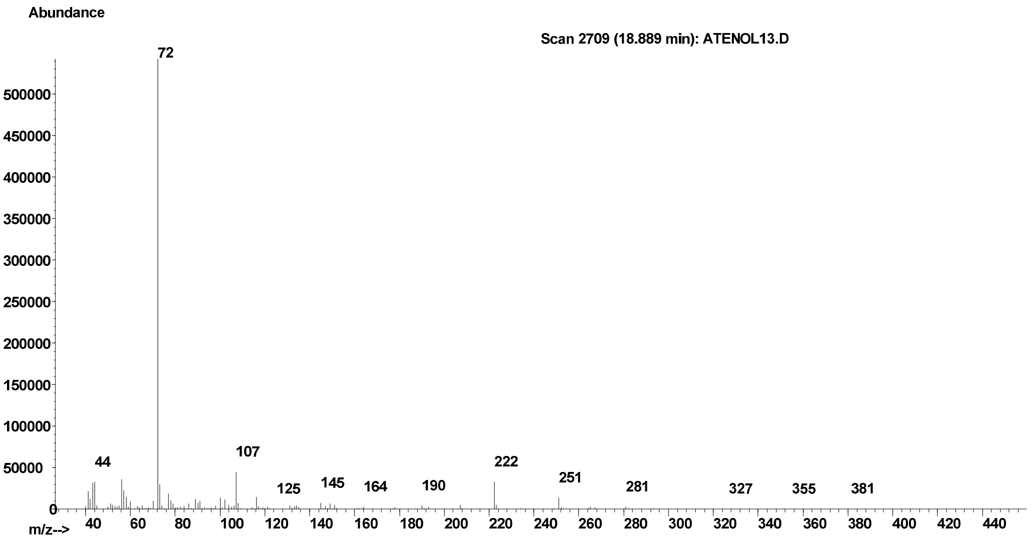

Figure SI 6. GC-MS analysis of recovered Atenolol after precipitation.

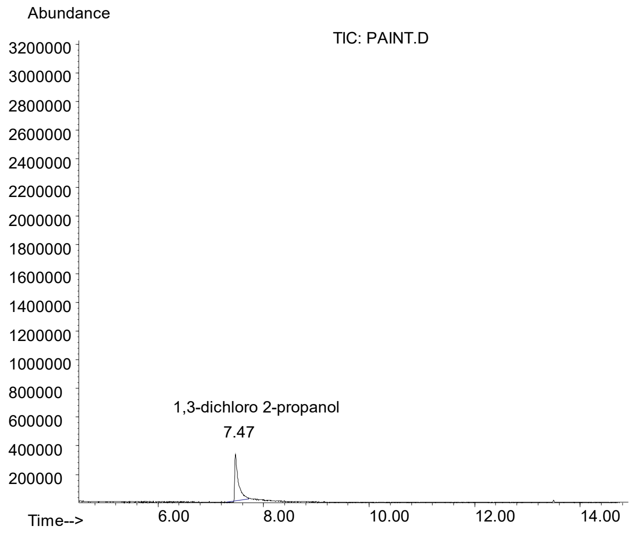

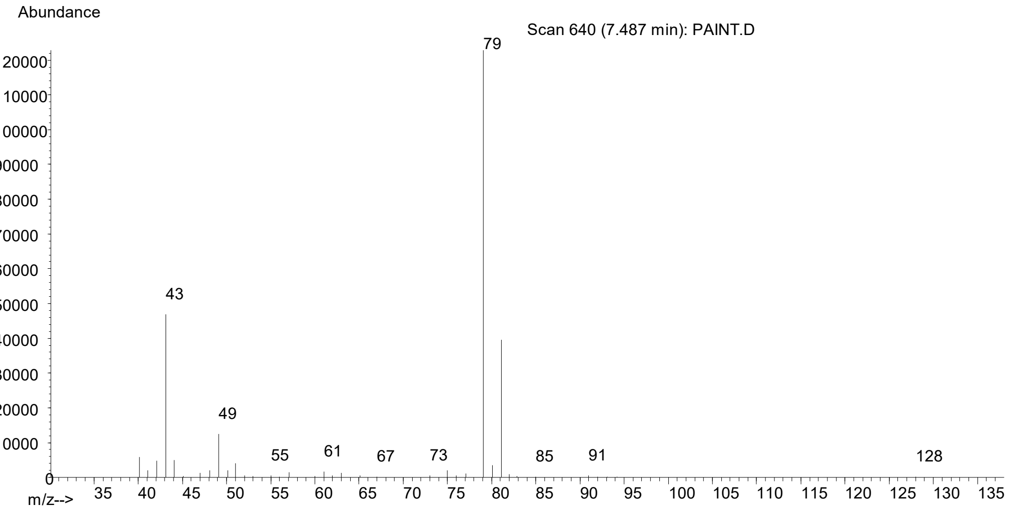

Figure SI 7. GC-MS analysis of the intermediate A

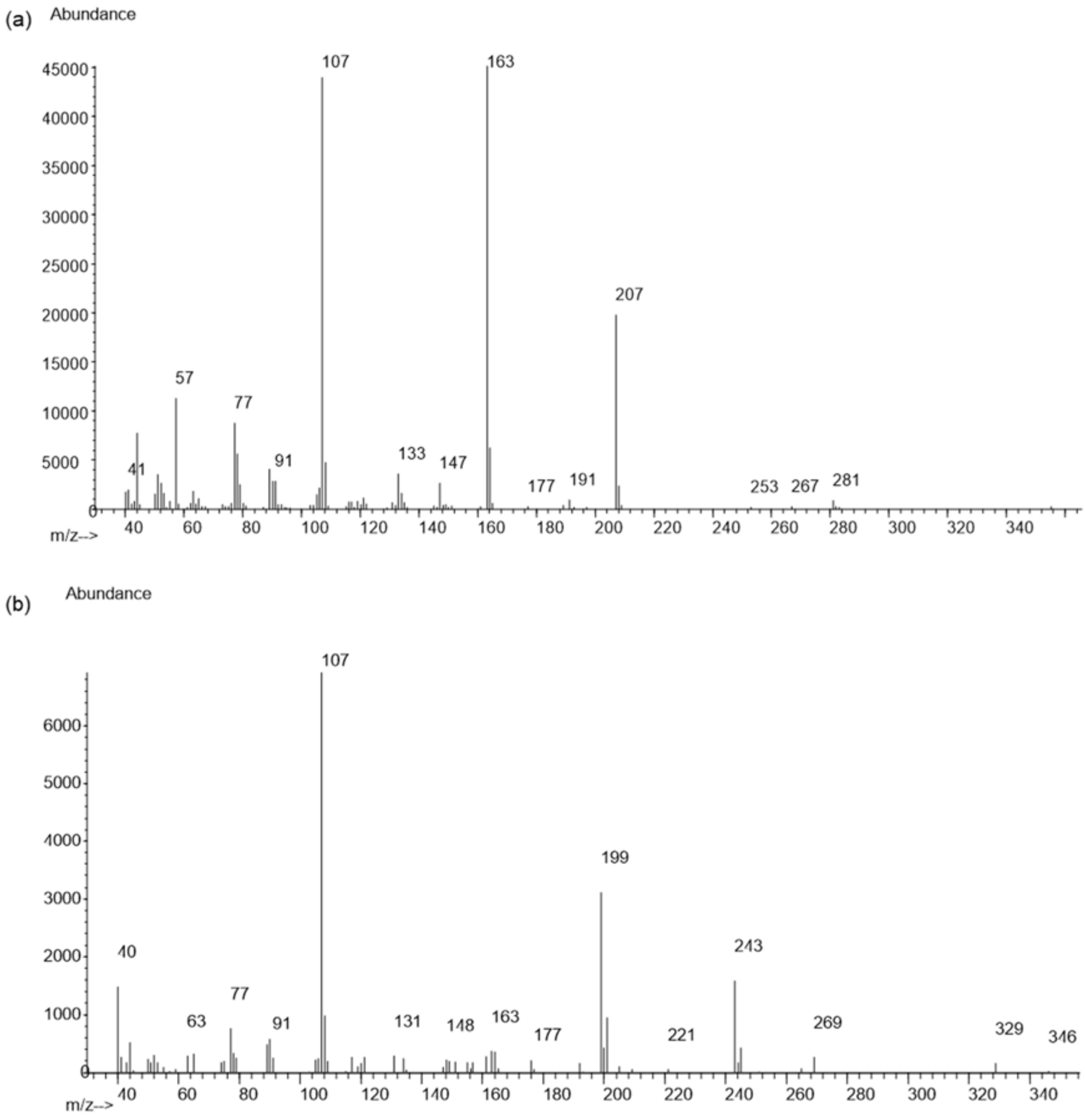

Figure SI 8. EI Mass spectra of (a) **3a** and (b) **3b**

8. Typical metrics applied at First Pass according to the CHEM21 Metrics Toolkit

our work

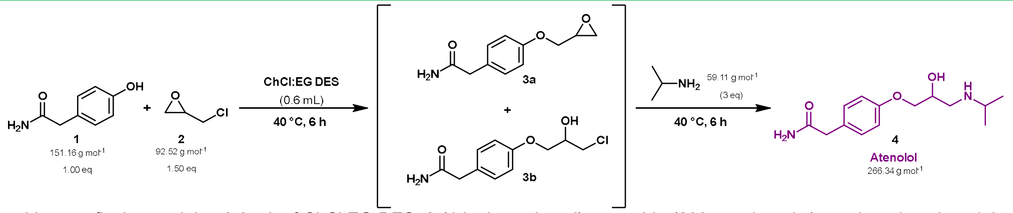

In a 25 mL round-bottom flask containing 0.6 mL of ChCl:EG DES, 2-(4-hydroxyphenyl)acetamide (200 mg, 1 equiv.) was introduced, and the mixture was stirred magnetically at 40 °C. Following the immediate solubilization of the amide, epichlorohydrin (0.184 g, 1.5 equiv.) was added dropwise, and the reaction mixture was further stirred at a temperature of 40 °C for 6 hours. The reaction progress was monitored by TLC and GC/MS. Upon completion, unreacted epichlorohydrin was removed by evaporation under reduced pressure. After removing the excess epichlorohydrin, the reaction mixture was further subjected to magnetic stirring and heated to a temperature of 40 °C. Subsequently, isopropylamine (0.235 g, 3 equiv.) was added dropwise. The reaction progress was monitored via TLC and GC/MS. Upon completion after 6 hours, the excess isopropylamine was removed by evaporation under reduced pressure. Atenolol recovery was performed by precipitation after addition of water. The precipitate was further washed with water and characterized by NMR analysis.

METRICS

Reactant (Limiting Reactant First)	Mass (g)	MW (g mmol^{-1})	equiv	Mol	Catalyst	Mass (g)	Reaction Solvent	Volume (mL)	Density (g.mL^{-1})	Mass (g)
2-(4-hydroxyphenyl)acetamide	0,200	151,16	1,00	0,00132			DES	0,60	1,12	0,67
epichlorohydrin	0,184	92,52	1,50	0,00198						
isopropylamine	0,235	59,11	3,00	0,00397						
Total	0,618	302,79		0,003308		0,000				0,67

Yield (%)	95		
AE (%)	88	OE	61,4
RME (%)	54		
MI total (g g^{-1})	3,9		
MI RRC (g g^{-1})	1,8		
MI Solvents (g g^{-1})	2,0	Conc (M)	2,21

	mass (g)	MW	mol
Product	0,33	266,34	0,00126

U.S. Patent 005290958A (1994)

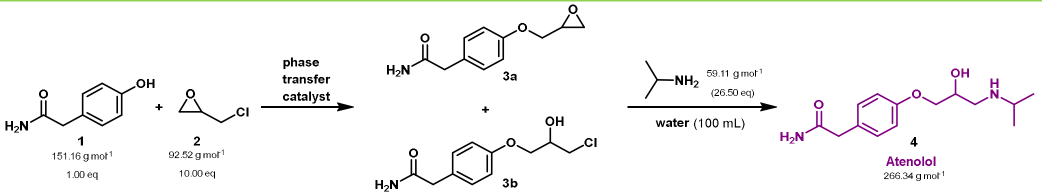

METRICS

Reactant (Limiting Reactant First)	Mass (g)	MW (g mmol^{-1})	equiv	Mol	Catalyst	Mass (g)	Reaction Solvent	Volume (mL)	Density (g.mL^{-1})	Mass (g)
2-(4-hydroxyphenyl)acetamide	9,977	151,16	1,00	0,06600	methyltridecylammonium chloride	0,4959	H_2O	100,00	1,00	100,00
epichlorohydrin	61,063	92,52	10,00	0,66000						
isopropylamine	103,383	59,11	26,50	1,74900						
Total	174,423	302,79		2,475000		0,496				100,00

Yield (%)	62		
AE (%)	88	OE	6,82
RME (%)	6		
MI total (g g^{-1})	25,2		
MI RRC (g g^{-1})	16,0		
MI Solvents (g g^{-1})	9,2	Conc (M)	0,66

	mass (g)	MW	mol
Product	10,90	266,34	0,04093

U.S. Patent 3663607(1972), 3836671(1974) and 3934032(1976); Kitaori *et al.* *Chem. Pharm. Bull.* 1998,46(3),505-507

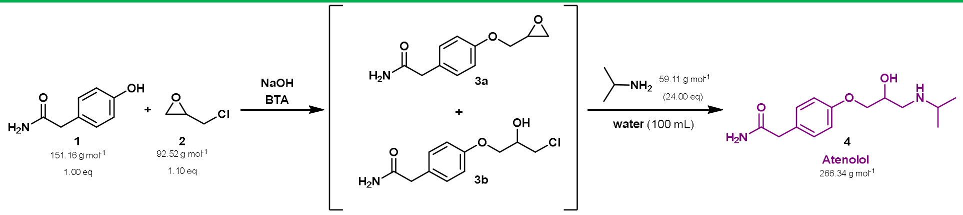

METRICS

Reactant (Limiting Reactant First)	Mass (g)	MW (g mmol^{-1})	equiv	Mol	Catalyst	Mass (g)	Reaction Solvent	Volume (mL)	Density (g.mL^{-1})	Mass (g)
2-(4-hydroxyphenyl)acetamide	35,70	151,16	1,00	0,236	benzyltrimethylammonium chloride	0,1800	H_2O	158,00	1,00	158,00
epichlorohydrin	24,04	92,52	1,10	0,260	NaOH	9,6000	H_2O	21	1	21,00
isopropylamine	335,05	59,11	24,00	5,668						
Total	394,78	302,79		6,164		9,780				179,00

Yield (%)	86		
AE (%)	88	OE	15,9
RME (%)	14		
MI total (g g^{-1})	10,8		
MI RRC (g g^{-1})	7,5		
MI Solvents (g g^{-1})	3,3	Conc (M)	1,495

	mass (g)	MW	mol
Product	53,90	266,34	0,202

Entry	AE (%)	RME (%)	OE (%)	PMI (g g^{-1})	PMI_{RRC} (g g^{-1})	PMI_{solv} (g g^{-1})
**our work**	**88**	**54**	**61,4**	**3,9**	**1,8**	**2,0**
U.S. Patent 005290958A (1994)	88	6	6,82	25,2	16,0	9,2
U.S. Patent 3663607(1972), 3836671(1974) and 3934032(1976); Kitaori *et al.* Chem. Pharm. Bull.1998,46(3),505-507	88	14	15,9	10,8	7,5	3,3

our work - Scale up 1 g

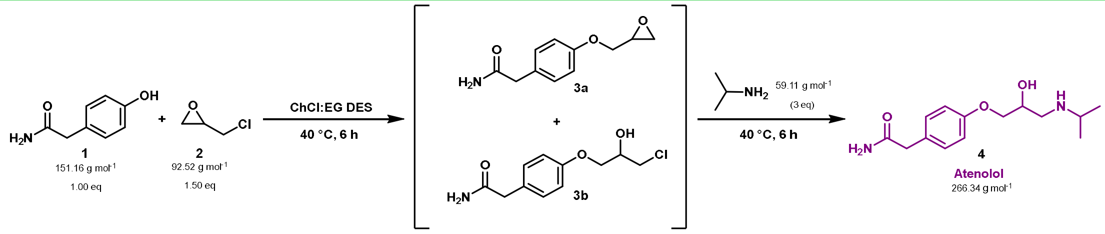

METRICS

Reactant (Limiting Reactant First)	Mass (g)	MW (g mmol^{-1})	equiv	Mol	Reaction Solvent	Volume (mL)	Density (g.mL^{-1})	Mass (g)
2-(4-hydroxyphenyl)acetamide	1,000	151,16	1,00	0,00662	DES	3,00	1,12	3,36
epichlorohydrin	0,918	92,52	1,50	0,00992				
isopropylamine	1,173	59,11	3,00	0,01985				
Total	3,091	302,79		0,016539				3,36

Yield (%)	95
AE (%)	88
RME (%)	54
MI total (g g^{-1})	3,9
MI RRC (g g^{-1})	1,8
MI Solvents (g g^{-1})	2,0

	mass (g)	MW	mol
Product	1,67	266,34	0,00628

Conc (M)	2,21

our work - Scale up 10 g

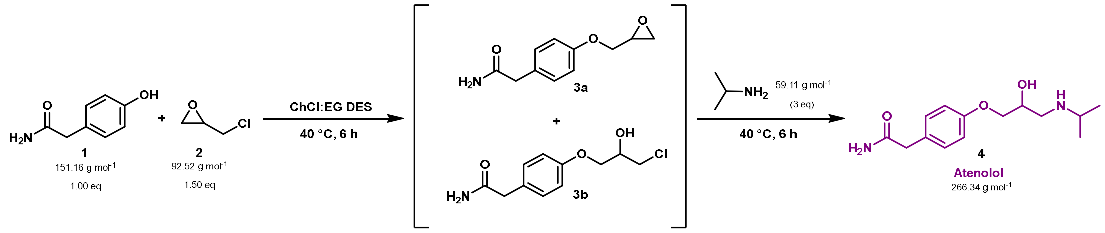

METRICS

Reactant (Limiting Reactant First)	Mass (g)	MW (g mmol^{-1})	equiv	Mol	Reaction Solvent	Volume (mL)	Density (g.mL^{-1})	Mass (g)
2-(4-hydroxyphenyl)acetamide	10,000	151,16	1,00	0,06616	DES	30,00	1,12	33,60
epichlorohydrin	9,181	92,52	1,50	0,09923				
isopropylamine	11,731	59,11	3,00	0,19847				
Total	30,912	302,79		0,165388				33,60

Yield (%)	95
AE (%)	88
RME (%)	54
MI total (g g^{-1})	3,9
MI RRC (g g^{-1})	1,8
MI Solvents (g g^{-1})	2,0

	mass (g)	MW	mol
Product	16,74	266,34	0,00629

Conc (M)	2,21

Mass Efficiency: PMI and E-factor

Reaction Steps

Step 01	
**Substrate**	mass (g)
2-(4-hydroxyphenyl)acetamide	0,2
**Reagents**	mass (g)
epichlorohydrin	0,184
**Organic Solvents**	mass (g)
DES	0,672
**Water or Aqueous Solutions**	mass (g)
**Desired Product**	mass (g)
intermediate X	0,246

Step 02	
**Substrate**	mass (g)
intermediate X	0,246
**Reagents**	mass (g)
isopropylamine	0,235
**Organic Solvents**	mass (g)
**Water or Aqueous Solutions**	mass (g)
water	5,0
**Desired Product**	mass (g)
ATENOLOL	0,300

**Material Inputs**	mass (g)
Substrate	0,2
Reagents	0,2
Solvents	0,7
Aqueous	0,0
Total	1,1
**Material Outputs**	mass (g)
Product	0,2
Waste	0,8

**Material Inputs**	mass (g)
Substrate	0,2
Reagents	0,2
Solvents	0,0
Aqueous	5,0
Total	5,5
**Material Outputs**	mass (g)
Product	0,3
Waste	5,2

**PMI**	**4**
**E-factor**	**3**

**PMI**	**18**
**PMI (cumulative)**	**21**
**E-factor**	**17**
**E-factor (cumulative)**	**20**

Process Mass Efficiency (PMI) and **Efficiency Factor** (E-factor) are material ratios.

PMI = total mass of inputs / mass of product. An ideal value is **1** (100% conversion of raw materials to product).

E-factor = mass of waste / mass of product. An ideal value is **close to 0** (i.e. no waste).

This calculation tool was brought to you by GreenChemWeb (www.greenchem.org)

We wish to acknowledge the ACS-GCI Pharmaceutical Roundtable for the concept of Process Mass Intensity and for producing the first calculation tool upon which this is based. Prof. Roger Sheldon of the Delft University of Technology is acknowledged for the concept of E-factor.
